# Designing Dynamic Stacked Bar Charts for Alarm Semantic Levels: Hierarchical Color Cues and Orientation on Perceptual Order and Search Efficiency

**DOI:** 10.3390/s25247589

**Published:** 2025-12-14

**Authors:** Jing Zhang, Qi Yan, Jinchun Wu, Weijia Ge

**Affiliations:** 1 College of Furnishings and Industrial Design, Nanjing Forestry University, Nanjing 210037, China; 15620260938@163.com (Q.Y.); geweijia@njfu.edu.cn (W.G.); 2 School of Digital Technology and Innovation Design, Jiangnan University, Fuqian 299, Jiangyin 214400, China; wjcseu@seu.edu.cn

**Keywords:** dynamic stacked bar charts (DSBCs), color cues, orientation, sensor interface, sensor-driven display interface

## Abstract

In sensor-based monitoring systems, the rapid and accurate recognition of alarm semantic levels is essential for maintaining operational reliability. Traditional static visualizations often fail to communicate these distinctions effectively under time pressure, whereas dynamic stacked bar charts (DSBCs) integrate multiple semantic layers into a compact, dynamic display. This study systematically investigated how color cues applied to auxiliary visual elements (background, foreground, labels, and scale lines) and chart orientation (horizontal vs. vertical) affect users’ alarm recognition performance. Thirty-two participants completed a semantic alarm recognition task involving DSBCs with various combinations of color-coded elements and orientations. Reaction time (RT) and accuracy (ACC) were analyzed using mixed-effects regression models. The results revealed that color cues in foreground and labels significantly enhanced both RT and ACC, whereas background and scale line color cues produced negligible effects. Orientation exerted a significant main effect on RT but not on ACC. Participants responded faster to horizontally oriented charts, indicating improved scanning efficiency. Moreover, increasing the number of color cues yielded higher ACC and shorter RTs, supporting a redundancy gain effect. However, no interaction was found between color cues and orientation, suggesting that these factors influence performance through distinct cognitive pathways. The findings align with theories of attentional guidance, redundancy gain, and spatial compatibility, and offer practical recommendations for alarm visualization design. Consequently, designers are advised to prioritize color coding of perceptually dominant elements, employ horizontal layouts in time-critical contexts, and implement redundant but non-overwhelming cues to enhance alarm recognition in complex sensor-based monitoring environments.

## 1. Introduction

**Figure 1:**
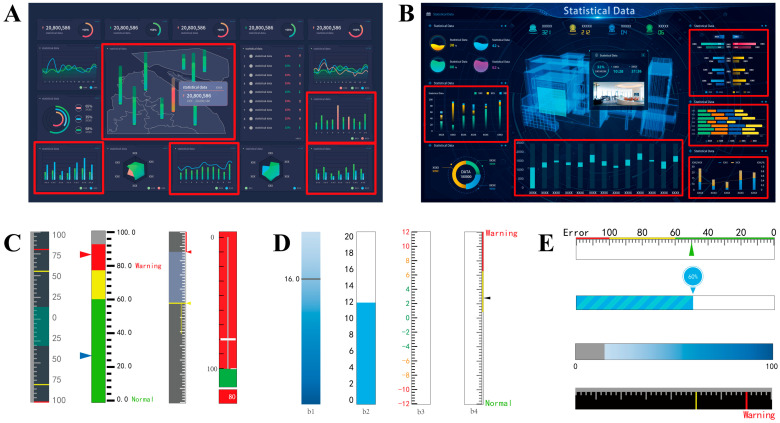
Examples of DSBCs. (**A**,**B**) are typical scenarios containing DSBCs (marked by red boxes). (**C**) Vertical dynamic stacked bar charts in the nuclear power plant. (**D**) Bar chart with a single-color cue, b1–b4 indicate color cues in background, foreground, labels and scale lines. (**E**) Horizontal bar chart.

In safety-critical monitoring environments that rely on sensor data, operators must rapidly and accurately identify different alarm semantic levels, such as normal, warning, and critical levels, to ensure timely intervention and maintain system reliability. Conventional static or simple bar charts often fail to convey these distinctions effectively under high cognitive load. In contrast, dynamic stacked bar charts (DSBCs) provide a compact and integrative visualization approach for simultaneously representing multiple semantic levels (see Figure 1). By transforming continuous sensor readings into intuitive hierarchical alarm cues, DSBCs bridge the gap between perceptual encoding and operational awareness. As a preattentive visual feature, color naturally draws attention to specific regions, and appropriate highlighting in dynamic displays thereby increases the likelihood of detecting alarm changes [1]. By employing distinct color coding across auxiliary visual elements such as background, foreground, text labels, and scale lines, DSBCs enable operators to perceive both the current system status and its corresponding threshold boundaries at a glance. For instance, in nuclear power plant interfaces, parameters such as temperature, pressure, and reactor power are frequently visualized using DSBCs, allowing operators to intuitively recognize the semantic differences among normal, warning, and critical conditions. Beyond the nuclear domain, DSBCs are also widely employed in other safety-critical systems, including aviation, high-speed railway, health-care dashboards, power generation plants, chemical process control, and industrial monitoring environments [2,3,4,5]. Given their extensive use and impact on situational awareness, the effective design and ergonomic optimization of DSBCs are crucial to ensuring both interpretative accuracy and operational efficiency in safety-critical contexts.

Despite their advantages, DSBCs pose several unresolved design challenges. First, although extensive research has demonstrated the benefits of color coding for visual search, existing studies have not addressed how color cues should be differentially assigned to various auxiliary visual elements (e.g., backgrounds vs. labels vs. scale lines) within DSBCs. Most prior work applies color globally rather than targeting specific elements, leaving it unclear whether different elements assume distinct and specialized roles in hierarchical alarm recognition. Unlike simple bar charts, DSBCs incorporate multiple auxiliary visual elements such as backgrounds, foregrounds, labels, and scale lines, each capable of conveying distinct color cues. However, the integration of multiple semantic thresholds makes color coding particularly complex, thereby increasing operators’ perceptual load and cognitive demands. From a color-coding perspective, relying on a single hue is insufficient to differentiate multiple categories adequately. Conversely, employing multiple hues or closely spaced color variations inevitably increases visual complexity and reduces color discriminability [6,7].

Second, DSBCs inherently combine dynamic and multi-level alarm conditions, yet much of the prior empirical evidence on color coding is derived from static or simplified stimuli. This limits the generalizability of earlier findings to DSBCs, where semantic thresholds evolve over time and impose stronger demands on perceptual grouping and cognitive processing. Consequently, it remains unclear whether previously documented color-coding effects generalize to dynamic multi-level alarm environments. Previous studies have demonstrated that poorly structured or inconsistent color strategies (e.g., ambiguous hues or insufficient contrast) can reduce visual salience and impair recognition efficiency [8].

Third, although the semantic hierarchy of alarms interacts with spatial layout, the influence of DSBC orientation (horizontal vs. vertical) on semantic-level perception has not been systematically examined. Orientation is often constrained by interface layouts, yet it fundamentally shapes perceptual order by influencing reading direction, grouping tendencies, and eye-movement patterns [9]. However, existing research provides only limited evidence on how these orientation-driven perceptual processes affect the detection of hierarchical alarm levels, particularly in dynamic DSBCs.

Building on the identified gaps in prior research on visualization and alarm perception, this study aimed to address the following research questions:

**RQ1:** How does the application of hierarchical color cues to different auxiliary elements of DSBCs (e.g., background, foreground, labels, scale lines) influence operators’ perceptual order and the recognition of multi-level alarm semantics?

**RQ2:** How does DSBC orientation (horizontal vs. vertical) affect the perception, ordering, and discrimination of hierarchical alarm levels, and does orientation interact with color cue placement?

**RQ3:** Under dynamic and evolving multi-level alarm conditions, do different color-coding strategies yield differential effects on visual search efficiency, semantic differentiation, and cognitive load?

This study makes three primary contributions to the design and understanding of dynamic stacked bar charts (DSBCs) in safety-critical monitoring environments. First, it provides the first systematic comparison of hierarchical color cue placement across multiple auxiliary visual elements (background, foreground, labels, and scale lines), revealing how each element differentially supports perceptual ordering and semantic-level discrimination. Second, it offers empirical evidence on how DSBC orientation (horizontal vs. vertical) influences the perception of multi-level alarm semantics and demonstrates that orientation effects operate independently of color cue placement. Third, by examining color-coding strategies under dynamic, evolving alarm conditions, it identifies how different color assignments affect visual search efficiency, cognitive load, and recognition accuracy. Collectively, these contributions establish an empirical foundation and provide design guidelines for optimizing DSBCs in safety-critical domains such as industrial control, nuclear power, aviation, and medical monitoring.

## 2. Related Work

### 2.1. Color Cues

Previous research has highlighted the importance of color coding in graphics and visualization design, demonstrating that color cues can substantially improve visual search efficiency and cognitive performance. A broad body of evidence has shown that color could significantly enhance recognition efficiency [10], facilitate the rapid identification of key information [11], and increase visual engagement [12]. Beyond these general benefits, studies have also revealed that color operates through layered attentional mechanisms. For example, Zhang et al. [13] found that color coding exerts layered effects on attention capture, suggesting that color cues applied to auxiliary elements may differentially influence performance. Likewise, color cues appear to be particularly advantageous under dynamic conditions. For instance, both Herman et al. [14] and Fu et al. [15] showed that color coding yields larger performance gains in dynamic than in static displays. Despite these insights, existing work has not yet clarified how color should be systematically controlled across different auxiliary visual elements.

A growing line of research further suggests that the effectiveness of color coding, whether influencing accuracy, response speed, attention allocation, or cognitive load, depends critically on its specific application within the visual display. Zhang et al. [13] demonstrated that color coding strategies significantly affect visual search performance and user experience. Classic visual search frameworks echo this view. Wolfe and Horowitz [16] emphasized that the diagnostic value of color is context-dependent, such that a color cue salient in one location (e.g., labels) may be negligible in another (e.g., backgrounds). Szafir [7] found that color discriminability varies with mark type and size, with elongated marks (e.g., bars) supporting greater discriminability than small point-like ones. Tangmanee and Ayutthaya [17] investigated how three display features of scale style, bar order, and bar width independently and interactively affect viewers’ perception bias of bar charts, directly demonstrating that auxiliary visual features are key variables influencing chart perception. Forrest et al. [18] conducted a meta-study on the “Grotta bars” used in observational neurology research and found that their presentation directly affects causal interpretation. Foreground–background relations present another dimension: foreground color cues in line drawings enable faster detection than background coloring [19], while background context itself can modulate color-signal detectability and introduce trade-offs with foreground cues [20]. Together, these findings converge on a key insight: although color is a powerful encoding tool, its benefits vary substantially across different auxiliary elements and design choices.

Notably, most of these paradigms have been developed using static or simplified visual formats, limiting their applicability to more complex and dynamic visualization types. In dynamic stacked bar charts (DSBCs), where multi-level thresholds evolve over time and perceptual demands shift continuously, the roles of auxiliary elements, such as backgrounds, foregrounds, labels, and scale lines, remain insufficiently understood. To date, both the main effects of these elements and their potential interactions with color cues remain underexplored, highlighting a critical gap that the present research aims to address.

### 2.2. Orientation Research

Orientation introduces an additional dimension of visual complexity to the dynamic stacked bar charts (DSBCs), influencing both their visual structure and perceptual processing. Although the role of orientation is often highlighted in visualization practice, empirical evidence for its effects remains limited and highly task-dependent. Eye-movement behavior research has demonstrated that orientation modulates scan paths and attentional allocation, with horizontal and vertical layouts eliciting distinct saccadic patterns and perceptual spans [21]. In interface and monitoring contexts, Wickens et al. [22] emphasized that display layout and sequencing directly affect the prioritization of alarms and status indicators, underscoring the critical role of perceptual order in effective monitoring. Similarly, Sarter [23] and Stanton et al. [24] argued that suboptimal orientation and ordering in aviation and control-room displays can delay the detection of critical alarms and increase operator workload. These findings collectively suggest that, in alarm semantic-level tasks, orientation likely determines the sequence of information processing.

However, despite its importance, the effects of orientation in DSBCs remain underexplored, especially regarding how alarm semantic levels are perceived and hierarchically ordered under different stacking orientations in complex monitoring contexts. Some studies suggest that horizontal layouts provide advantages under time pressure. For instance, Thornton et al. [25] found that horizontal targets can be located more efficiently in dynamic search tasks. In contrast, vertical stacking has been shown to facilitate quantitative information extraction under interference conditions [26]. Moreover, design recommendations indicate that ordering conventions differ substantially between horizontal and vertical charts, with implications for maintaining semantic consistency and interpretability [27]. Comparative visualization studies further suggest that the arrangement of visual marks strongly shapes perceptual order and determines which information is initially attended to, indicating that orientation and sequencing are inseparable design variables [28]. Taken together, these findings suggest that chart orientation is closely related to perceptual order, task demands, and attentional resources, and may even interact with color cues to influence alarm perception. Nevertheless, the effects of orientation in DSBCs, particularly regarding how alarm semantic levels are perceived and ordered, remain insufficiently addressed in experimental research.

Based on the above analysis, this study employed a behavioral experimental approach to investigate how color cues assigned to auxiliary visual elements (background, foreground, labels, scale lines) and stacking orientation influence the recognition of semantic alarm levels in dynamic stacked bar charts (DSBCs). Two experimental factors were systematically manipulated: (1) the application of color cues across auxiliary visual elements (background, foreground, labels, and scale lines), and (2) the stacking orientation of the chart (horizontal vs. vertical).

The experimental stimuli consisted of a series of newly designed DSBCs developed to simulate graded alarm visualization tasks. In each trial, three blank stacked bar charts were first presented at the center of the screen for 1000 ms, followed by a dynamic update that displayed three alarm states (normal, warning, and critical) in randomized proportions and spatial arrangements. Participants were instructed to sequentially identify and click the chart regions corresponding to the normal (green), warning (yellow), and critical (red) alarm levels. By systematically measuring perceptual order and search efficiency under different combinations of hierarchical color cues and orientation, this study aims to establish effective visual encoding strategies for DSBCs. The findings are expected to enhance the interpretability and perceptual clarity of alarm information in dynamic visualizations and to provide empirical guidance for the ergonomic design of complex monitoring interfaces in safety-critical systems.

## 3. Method

### 3.1. Participants

Thirty-two undergraduate students (14 males and 18 females; mean age M = 21.88, SD = 3.02) from Nanjing Forestry University were recruited. All participants were right-handed, had prior experience with computer-based tasks, and demonstrated a clear understanding of how chart elements represented different status conditions. All were also required to have visual acuity (uncorrected or corrected to normal) of at least 1.0, as measured by the International Standard Visual Acuity Scale. Participants were screened for congenital color-vision deficiencies using the Ishihara 24-plate test administered under standard daylight illumination [29]. No participant reported any form of color vision deficiency. All participants provided written informed consent before the experiment. Each participant received a small gift (valued at approximately ¥25) as compensation.

### 3.2. Experimental Design

This study employed a within-subjects factorial design to examine how hierarchical color cues and chart orientation influence semantic alarm recognition. Five independent variables were manipulated: Background Color Cue (presence vs. absence), Foreground Color Cue (presence vs. absence), Label Color Cue (presence vs. absence), Scale Line Color Cue (presence vs. absence), and Stacking Orientation (horizontal vs. vertical). These variables formed a 2 (Background) × 2 (Foreground) × 2 (Label) × 2 (Scale Line) × 2 (Orientation) factorial structure. All participants completed trials in every condition. The dependent measures were reaction time (RT) and accuracy (ACC) in identifying the semantic alarm level displayed in each dynamic stacked bar chart (DSBC). Each trial presented a DSBC that transitioned through the system’s semantic alarm levels (normal, warning, critical). Two additional control conditions, a default color cue and a blank state, were included to ensure stable baselines for comparison. The presentation order was randomized across four possible state-transition sequences.

### 3.3. Experimental Materials

**Figure 2:**
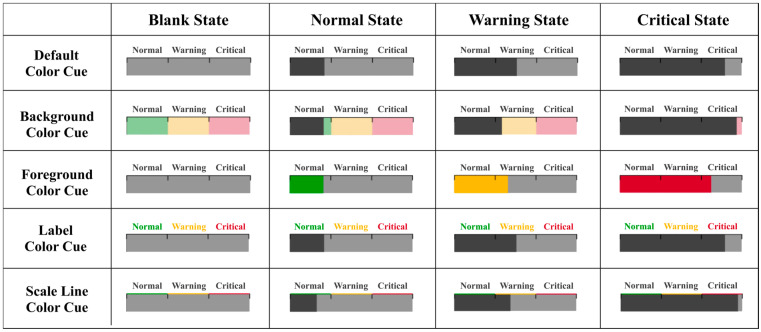
Examples of dynamic stacked bar charts (DSBCs) with a horizontal stacking orientation.

The stimuli consisted of dynamic stacked bar charts (DSBCs) specifically designed for this study. The stimulus set was evaluated for accessibility using contrast ratio checks to ensure discriminability. Each chart displayed one system’s state, one blank state or one of the three operational states: normal, warning, or critical (see Figure 2). The visual appearance of each stimulus was determined by the color-cue manipulations defined in the experimental design. Color coding followed standard alarm semantics. For color coding conditions, green (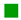 RGB: #039B00; CIE LAB: L56/a-54/b56) denoted the normal state, yellow (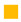 RGB: #FFBA00; CIE LAB: L80/a17/b82) represented the warning state, and red (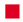 RGB: #BF0029; CIE LAB: L41/a66/b37) indicated the critical state. The red-yellow-green (RYG) scheme was adopted because it is the dominant standard in many safety-critical domains (e.g., industrial control, nuclear operations, aviation, medical monitoring). In non-color-cue conditions, the background was set to light gray (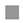 RGB: #969696; CIE LAB: L62/a0/b0) and the foreground and auxiliary elements to dark gray (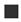 RGB: #434343; CIE LAB: L28/a0/b0). When background color cues were present, the corresponding alarm color was applied at 30% opacity to maintain adequate contrast with other visual elements. Foreground, label, and scale line cues were applied by rendering those elements directly using the appropriate alarm color. All stimuli were animated using identical timing parameters and displayed at a consistent size and resolution to ensure comparability across conditions.

### 3.4. Experimental Task

**Figure 3:**
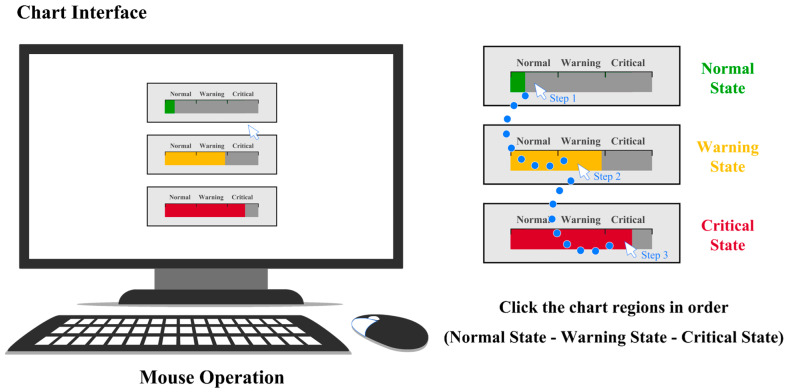
Experimental scene and stimuli.

The experimental task was a semantic alarm recognition task, designed to evaluate participants’ accuracy and visual search performance in identifying target states (normal, warning, critical) and performing corresponding actions under different visual encoding conditions. During the experiment, participants were instructed to sequentially select the chart regions representing the three system states: normal state, warning state, and critical state (see Figure 3). To control for potential sequence effects in the spatial sequencing of alarm information, the presentation order of the three alarm levels was systematically randomized across trials.

### 3.5. Experimental Procedure

The experiment was conducted in the Human–Computer Interaction Laboratory at Nanjing Forestry University under controlled ambient lighting conditions (approximately 300 lx). All stimuli were presented, and participant responses were recorded using a custom experimental program developed in E-Prime 3.0. The program was executed on a laptop equipped with a 15.6-inch LCD monitor (1920 × 1080 pixels; 144 Hz refresh rate). The viewing distance was maintained between 50 cm and 60 cm for all participants throughout the experimental session.

**Figure 4:**
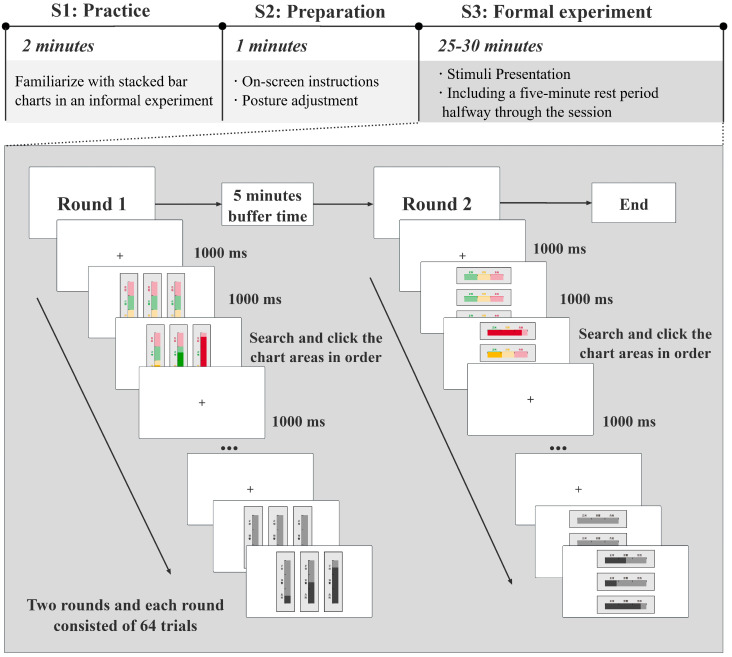
Experimental procedure.

Upon arrival, participants provided basic demographic information. Prior to the formal experiment, each participant completed five practice trials to ensure comprehension of the task and familiarity with the interface. During the formal session, combinations of five independent variables (background color cue, foreground color cue, label color cue, scale line color cue, and stacking orientation) and control variables were presented in a randomized order to minimize potential order and carryover effects. Each trial began with a central fixation cross that appeared for 1000 ms to focus participants’ attention, followed by the presentation of three blank stacked bar charts for 1000 ms. The charts then displayed three alarm states (normal, warning, critical), in randomized proportions and spatial arrangements. Participants were instructed to click the corresponding chart regions sequentially in the prescribed order (normal → warning → critical). Each state remained onscreen until a response was made. No time limit was imposed, and participants could respond without penalty. Each trial ended with a 1000 ms fixation cross before the onset of the next stimulus. The only dynamic visual event in the experiment was the presentation of each new stimulus, which appeared through a discrete static refresh triggered by a change in the monitored state. No other motion-based animations were used, and all visual elements remained static throughout the presentation of each stimulus. The experiment consisted of two rounds, each containing 64 randomized trials, resulting in 128 trials per participant. Each condition was presented in 4 trials per participant. Previous perceptual and HCI studies using similar factorial designs have shown that 3–6 repetitions per condition are generally sufficient when stimuli are highly controlled [30]. Thus, the number of trials ensured adequate power for detecting group-level effects. A 5 min rest period was provided between rounds. The total duration of the experimental session was approximately 30 min per participant. The experimental procedure is illustrated in Figure 4.

### 3.6. Data Collection and Analysis

Reaction time (RT) and accuracy (ACC) during the visual search task were recorded for subsequent statistical analyses. Prior to analysis, invalid trials and statistical outliers were excluded. Due to data preservation issues during the experimental process, data from two participants were removed, resulting in 30 valid datasets for subsequent analyses. Overall, participants demonstrated high accuracy (mean ACC = 92.6%). For RT analyses, only correct trials were retained. Trials with RTs exceeding three median absolute deviations (MADs) from each participant’s median RT were excluded as extreme outliers. Statistical analyses were performed using R Studio 4.4.1 and SPSS 26.0. Specifically, linear mixed-effects regression models and logistic regression analyses were employed to examine the effects of the independent variables on RT and ACC, respectively. Additionally, analysis of variance (ANOVA) was conducted to assess the main and interaction effects of the color-coding factors and relevant covariates. Statistical significance was set at *p* < 0.05.

## 4. Results

### 4.1. Behavioral Performance of Color Cues of Dynamic Complex Stacked Bar Charts (DSBCs)

#### 4.1.1. Color Cues

**Figure 5:**
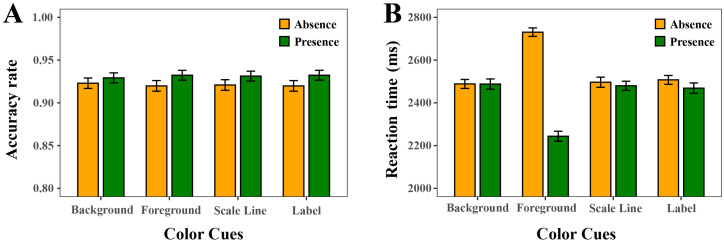
Mean accuracy rate (ACC) and reaction time (RT) under color-coded versus non-color-coded conditions for the background, foreground, scale line, and label. (**A**) ACC. (**B**) RT.

The statistical results (see Figure 5, Table 1, Table 2 and Table 3) revealed a significant main effect of Foreground Color Cue on ACC (ΔAIC = −3, LLR χ^2^(1) = 5.0044, *p* < 0.05) and on RT (ΔAIC = −470, LLR χ^2^(1) = 472.4467, *p* < 0.001). Specifically, participants achieved higher accuracy (ACC) and lower reaction time (RT) in the color coding condition than in the non-color coding condition, indicating that the Foreground Color Cue improved both response efficiency and recognition reliability. Moreover, RT analysis revealed a strong effect of foreground color coding, namely, participants responded significantly faster when the foreground bars were color-coded. The statistical results also revealed a significant main effect of text Label Color Cue on visual search performance, reflected in ACC (ΔAIC = −3.05, LLR χ^2^(1) = 5.0478, *p* < 0.05) and reduced RT (ΔAIC = −4, LLR χ^2^(1) = 6.0828, *p* < 0.05), while ACC remained consistently high across both conditions. However, Background Color Cue showed no significant effect on either ACC (ΔAIC = 0.74, LLR χ^2^(1) = 1.2555, *p* = 0.263) or RT (ΔAIC = 2, LLR χ^2^(1) = 0.2137, *p* = 0.644). Similarly, the Scale Line Color Cue condition showed no significant effect on ACC (ΔAIC = −1.53, LLR χ^2^(1) = 3.5331, *p* = 0.060) and RT (ΔAIC = 0, LLR χ^2^(1) = 1.3369, *p* = 0.248), although the effect on ACC approached significance.

#### 4.1.2. Effect Size of RT and ACC

To further quantify the magnitude of the observed mean differences, both partial eta squared (*η_p_*^2^) and Cohen’s d values were computed based on model variance components [31]. The results revealed a large effect of Foreground Color Cue (*η_p_*^2^ = 0.14, d = 0.79, 95% CI [0.72, 0.86]), indicating a strong influence. In contrast, the effects of Background Color Cue (*η_p_*^2^ < 0.001, d = 0.01, 95% CI [–0.05, 0.08]), Label Color Cue (*η_p_*^2^ = 0.002, d = 0.09, 95% CI [0.02, 0.15]), and Scale Line Color Cue (*η_p_*^2^ < 0.001, d = 0.04, 95% CI [–0.03, 0.11]) were small to negligible. According to conventional benchmarks [32], only the Foreground Color Cue condition produced a practically meaningful effect, whereas the remaining factors accounted for relatively little variance.

To complement the RT analysis, a generalized linear mixed-effects model (GLMM) with a binomial link was used to examine ACC. Effect sizes were expressed as odds ratios (ORs) with 95% confidence intervals. The results revealed a significant main effect of Foreground Color Cue (*p* = 0.020, OR = 0.65, 95% CI [0.46, 0.94]) and Label Color Cue (*p* = 0.021, OR = 0.61, 95% CI [0.42, 0.90]), indicating that both factors reliably influenced response accuracy. In contrast, neither Background Color Cue (*p* = 0.244, OR = 0.81, 95% CI [0.57, 1.15]) nor Scale Line Color Cue (*p* = 0.053, OR = 0.70, 95% CI [0.49, 1.00]) reached significance. Although Foreground Color Cue and Label Color Cue showed statistically significant effects, the corresponding standardized proportion differences (Cohen’s h < 0.20) indicate that their practical magnitudes were small, consistent with the RT findings.

#### 4.1.3. Interaction Effects

For ACC, a significant interaction was observed between Background Color Cue and Scale Line Color Cue (ΔAIC = −5.5, LLR χ^2^(1) = 7.4946, *p* < 0.01). A significant interaction was also found between Label Color Cue and Scale Line Color Cue (ΔAIC = −2.7, LLR χ^2^(1) = 4.6993, *p* < 0.05). For RT, the most robust interaction occurred between Foreground Color Cue and Background Color Cue (ΔAIC = −5, LLR χ^2^(1) = 7.3854, *p* < 0.01). Moreover, there was a significant interaction between Background Color Cue and Scale Line Color Cue (ΔAIC = −3, LLR χ^2^(1) = 5.7907, *p* < 0.05). No other two-way interactions reached significance. The analysis of four-way interactions demonstrated a divergence between ACC and RT outcomes. For ACC, the four-way interaction among Background Color Cue, Scale Line Color Cue, Label Color Cue, and Foreground Color Cue was not significant (ΔAIC = 2.19, LLR χ^2^(1) = 7.8154, *p* = 0.167). In contrast, the RT analysis revealed a significant four-way interaction among Background Color Cue, Scale Line Color Cue, Label Color Cue, and Foreground Color Cue (ΔAIC = −39, LLR χ^2^(1) = 49.2977, *p* < 0.001).

Following the significant four-way interaction observed for RT, additional post hoc analyses were conducted to clarify the structure of this higher-order effect. A series of nested linear mixed-effects models was fitted, sequentially adding each of the four possible three-way interactions. For each comparison, likelihood-ratio tests and ΔAIC values were examined to quantify the contribution of the added interaction term. As shown in Table 4, the four three-way interactions exhibited a non-convergent pattern: some improved model fit (e.g., Background Color Cue × Label Color Cue × Scale Line Color Cue, ΔAIC = −3; Foreground Color Cue × Background Color Cue × Scale Line Color Cue, ΔAIC = −14), one worsened the model (Foreground Color Cue × Background Color Cue × Label Color Cue, ΔAIC = +3), and one produced a very large improvement (Foreground Color Cue × Label Color Cue × Scale Line Color Cue, ΔAIC = −33). This inconsistency in both magnitude and direction indicates that no subset of three-way interactions accounts for the four-way effect. Instead, the significant four-way interaction likely reflects a cumulative, non-linear reorganization of attentional allocation that emerges only when all four cues co-occur.

### 4.2. Behavioral Performance of Orientation of the Dynamic Complex Stacked Bar Charts (DSBCs)

#### 4.2.1. Orientation

**Figure 6:**
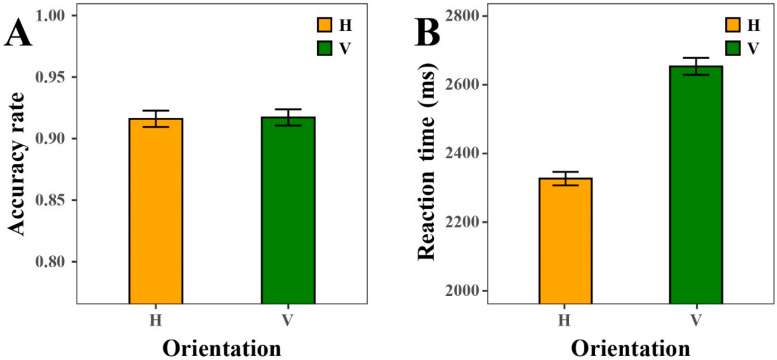
The ACC and RT under horizontal and vertical orientation conditions. (**A**) ACC. (**B**) RT.

**Figure 7:**
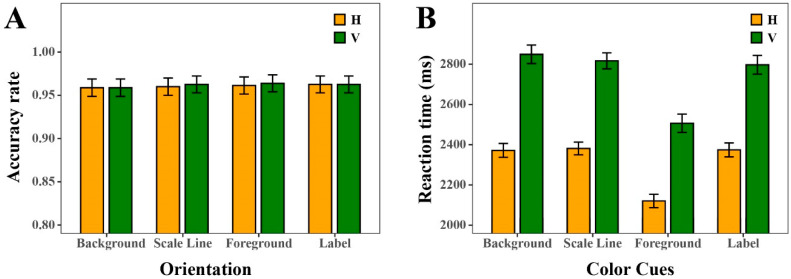
The ACC and RT of Background Color Cue, Foreground Color Cue, Scale Line Color Cue, and Label Color Cue with horizontal and vertical orientation. (**A**) ACC. (**B**) RT.

The statistical results (see Figure 6 and Figure 7) revealed that the orientation of DSBCs did not produce significant differences in ACC, indicating that participants’ performance was stable across both horizontal and vertical displays. In contrast, orientation exerted a significant influence on RT, with regression analyses confirming a statistically significant effect (ΔAIC = −178, LLR χ^2^(1) = 179.9070, *p* < 0.001). RTs were consistently lower in the horizontal orientation than in the vertical orientation, and this advantage persisted across all four types of color cues (Background Color Cue, Foreground Color Cue, Scale Line Color Cue, and Label Color Cue). The aggregated comparison further underscores a pronounced orientation effect: participants responded markedly faster when charts were presented horizontally, whereas vertical orientation systematically imposed greater processing demands.

To further clarify whether the observed four-way interaction in RT might have been driven by dependencies between orientation and the color-related cues, we conducted an additional set of nested mixed-effects models examining the four orientation × color-cue interactions (see Table 5 and Table 6). For ACC, none of the interactions between orientation and the four color cues (all *p* > 0.05) reached significance. For RT, only the Orientation × Foreground Color Cue interaction was significant (ΔAIC = −488, LLR χ^2^(2) = 492.781, *p* < 0.001), whereas the interactions with scale-line color, label color, and background color were much weaker, yielding only very small improvements in model fit (ΔAIC = −3 in both cases) and modest chi-square values (7.24 and 6.59, respectively). This pattern indicates that orientation does not form a coherent or systematic interaction structure with the color cues and therefore cannot account for the four-way color interaction observed in RT. Despite a strong main effect of orientation, its interactions with the four color cues were not consistent across dependent measures or color dimensions, indicating that orientation does not systematically interact with the color attributes.

#### 4.2.2. Effect Size of RT and ACC

Similarly, to further quantify the magnitude of the observed mean differences, Cohen’s d values were computed based on model variance components. The results for the orientation factor revealed a medium effect size in RT (d = −0.47, 95% CI [−0.53, −0.40]), indicating participants responded faster in the horizontal than in the vertical orientation. However, a negligible effect on ACC (OR = 0.97, 95% CI [0.68, 1.37]) was observed, suggesting this speed advantage did not compromise response reliability.

### 4.3. Behavioral Performance of Cue Number and Cue Type

#### 4.3.1. Effects of Cue Number

**Figure 8:**
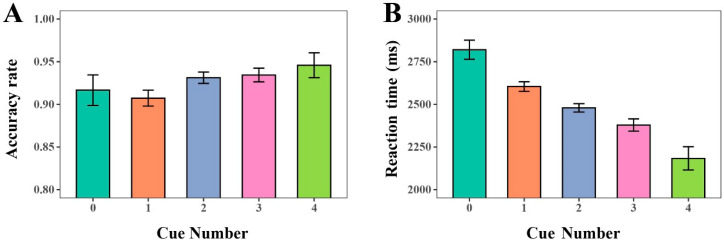
The ACC and RT of the cue number. (**A**) ACC. (**B**) RT.

**Figure 9:**
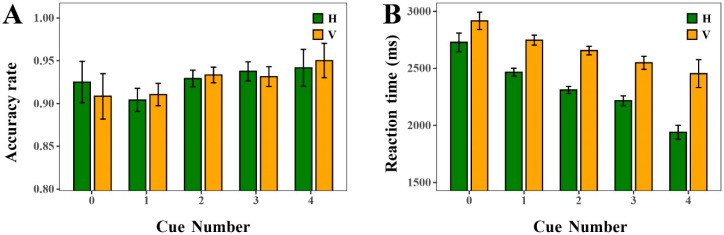
The ACC and RT of different cue numbers with horizontal and vertical orientation. (**A**) ACC. (**B**) RT.

Logistic mixed-effects regression results indicated a significant effect of cue number on ACC (ΔAIC = −10.37, LLR χ^2^(4) = 18.3728, *p* = 0.01), and linear mixed-effects regression results revealed a significant effect on RT (ΔAIC = −151, LLR χ^2^(4) = 158.729, *p* < 0.001). ACC increased gradually with the number of cues, ranging from 0.89 in the no-cue condition to 0.96 in the four-cue condition (see Figure 8). Also, the results showed consistently high ACC across horizontal and vertical orientations (see Figure 9A). Regarding RT, the results revealed that RTs decreased systematically as cue number increased, from approximately 2800–3000 ms in the no-cue condition to below 2000 ms with four cues (see Figure 9B). Further statistical tests revealed that performance differences across cue-number conditions reached significance in certain comparisons. For instance, under the horizontal orientation, ACC was significantly higher when three cues were provided compared to when only one cue was available (contrast estimate OR ≈ 3.0, *p* < 0.05). Although ACC with four cues showed a marginal increase relative to three cues, this difference was not statistically significant. RT was further reduced, suggesting diminishing returns.

#### 4.3.2. Effects of Cue Type

**Figure 10:**
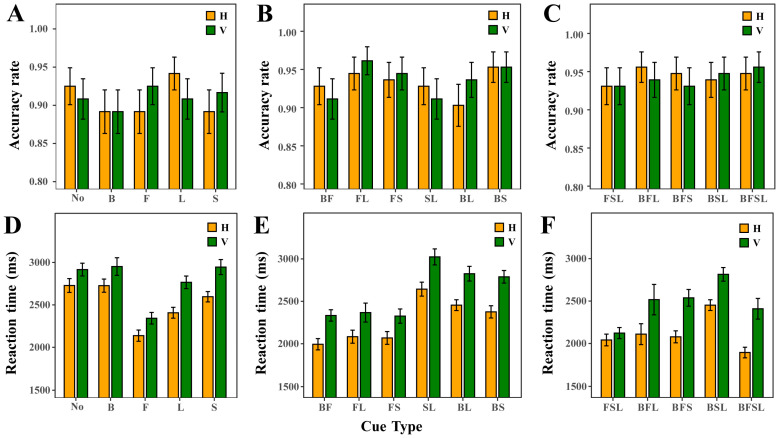
ACC and RT of the combination of different cues with horizontal and vertical orientation. (**A**–**C**) ACC for cue combinations. (**D**–**F**) RT for cue combinations. B denotes Background Color Cue. F denotes Foreground Color Cue. L denotes Label Color Cue. S denotes Scale Line Color Cue.

The results of Cue Types (see Figure 10) indicate marked differences in the promoting effects of different cues under single-cue conditions. Under the vertical orientation, the use of Foreground Color Cue alone yielded the highest ACC (accuracy ≈ 0.91) and produced the shortest RTs among all single-cue conditions in this orientation (RT ≈ 2350 ms). Under the horizontal orientation, the use of Label Color Cue alone produced the highest ACC, maintaining a relatively high level (accuracy ≈ 0.94), whereas Foreground Color Cue alone resulted in the shortest RTs within this condition group (RT ≈ 2100 ms).Under dual-cue combination conditions, complementary effects emerged between different cues. All dual-cue combinations that included Foreground Color Cue (e.g., Foreground Color Cue + Label Color Cue, Foreground Color Cue + Scale Line Color Cue, Background Color Cue + Foreground Color Cue) led to consistently shorter RTs compared with the No-Color-Cue condition in both horizontal and vertical orientations, and their ACC were consistently higher than those observed in No-Color-Cue conditions.When triple-cue were applied simultaneously, performance approached a ceiling level. In both horizontal and vertical orientations, all triple-cue combinations yielded higher ACC than No-Color-Cue condition, and RTs were consistently shorter. Among the triple-cue conditions, the combination excluding Background Color Cue (Foreground + Scale Line + Label) produced the shortest RTs. The all-cue condition, the horizontal orientation yielded the lowest RTs overall, with ACC slightly lower than that of the triple-cue combination excluding Background Color Cue (Foreground Color Cue + Scale Line Color Cue + Label Color Cue), whereas under the vertical orientation, ACC reached its highest level.

## 5. Discussion

This study examined two underexplored visual encoding strategies in dynamic stacked bar charts (DSBCs): color coding of auxiliary visual elements (Background, Foreground, Labels, Scale Lines), and stacking orientation as a spatial encoding strategy (horizontal vs. vertical orientation). By systematically measuring perceptual order and search efficiency under different combinations of hierarchical color cues and orientation encoding, this study aimed to offer effective visual encoding strategies for DSBCs in dynamic visualizations and to provide empirical guidance for the ergonomic design of complex monitoring interfaces in safety-critical systems.

### 5.1. Effects of Hierarchical Color Cues on Perceptual Order and Semantic Recognition (RQ1)

The behavioral results indicated that color cues applied to the foreground and labels significantly improved recognition performance. Compared with the non-color coding condition, participants achieved higher accuracy (ACC) and shorter reaction times (RTs) when these elements were color-coded. This result is consistent with attentional guidance theory, which posits that pre-attentive color signals can effectively direct visual attention toward critical information, thereby reducing search costs [33,34]. One possible explanation concerns the semantic dimension of color-meaning mappings, which may account for the particularly strong effects observed for label text. Prior studies have demonstrated that color-meaning associations are constrained by semantic discriminability [35], suggesting that similar asymmetries may exist across different visual elements within stacked bar charts [36]. Specifically, differences in semantic salience between textual labels and graphical regions could explain why color cues in the foreground and labels were more effective than those applied to backgrounds and scale lines [36,37]. Another explanation might relate to the number of color cues. In the background-color condition, the display simultaneously presented three alarm colors, while the stacked bars remained gray, resulting in more than four distinct colors in the chart. In contrast, the foreground-color condition typically involved only two colors (one for the background and one for the stacked bars). While moderate redundancy in visual cues can enhance recognition, excessive color coding may reduce perceptual efficiency [10]. Redundant color coding could decrease user engagement, and increasing the number of hues could raise cognitive load, thereby reducing efficiency in high-demand tasks [6,38]. Similarly, previous studies have also shown that inconsistent or redundant color cues can impair learning and behavioral performance [39]. Empirical evidence from visual search tasks supports this view. For instance, Giovannangeli et al. [40] reported that performance declines significantly once the number of color categories exceeds a perceptual threshold, reflecting the limited capacity of the human visual system to process multiple colors simultaneously. Collectively, our findings provide evidence that increasing the number of color cues improves alarm recognition performance, as redundancy both amplifies attentional guidance and reduces decision uncertainty, leading to higher accuracy and faster responses. This interpretation is also supported by Martinovic et al. [41] and Friedrich and Vollrath [42].

In the present study, the foreground cue differed substantially from other cue types in its visual form, and these differences help explain the observed reaction time advantages. Specifically, in the foreground non-color condition, the foreground region was rendered in a dark gray tone against a light gray background. The limited contrast between these two gray levels resulted in a weak luminance boundary, reducing the visual salience of the foreground. Owing to its presentation as a large, filled surface rather than a thin linear element (e.g., scale lines or text labels), the foreground cue carried substantially greater visual weight. Consequently, in the non-color condition, participants could not rely on chromatic coding and were forced to distinguish regions solely based on subtle grayscale differences. This placed higher demands on low-level visual segmentation mechanisms and consequently increased both visual-search load and decision time.

The diminished contrast and blurred region boundaries likely required participants to engage in additional feature integration and more deliberate allocation of attentional resources when identifying the target bar segment. Such processes naturally prolong reaction time. This account aligns with classic visual search theories, including Feature Integration Theory and the Guided Search model [43,44], which posit that in the absence of salient guiding features, search becomes more serial and fine-grained, thus yielding longer reaction times (RTs).

### 5.2. Effects of DSBC Orientation and Its Interaction with Color Cues (RQ2)

The behavioral results revealed that reaction time (RTs) were consistently shorter in the horizontal orientation compared with the vertical orientation, an advantage observed across all four types of color cues. No significant differences in accuracy (ACC) were found between the two orientations. This pattern suggests that a horizontal orientation layout facilitates faster information processing and more efficient visual scanning without compromising accuracy. A likely explanation is that horizontal arrangements align with natural visual scanning habits. In many reading cultures, left-to-right horizontal scanning is the default, enabling smoother perceptual transitions between adjacent visual elements. Eye-movement research provides strong evidence for this interpretation. For instance, Rayner [45] showed that horizontal scanning dominates reading and information processing, and that this habit extends to non-textual visual tasks. Vertical orientation layout, in contrast, requires top-to-bottom sequential scanning, which can interrupt perceptual continuity and impose additional cognitive load. This interpretation is consistent with prior findings in display design and visual ergonomics. Ware and Arsenault [46] emphasized that spatial alignment with natural scanning direction enhances scanning speed and reduces cognitive effort, particularly in time-sensitive monitoring contexts. A complementary explanation involves perceptual organization and feature integration. Wolfe and Horowitz [16] argued that visual search performance depends on the diagnosticity of features and their spatial arrangement. Displays that conform to habitual perceptual strategies facilitate more efficient allocation of attentional resources, whereas those that conflict with these strategies increase search time. Similarly, Wickens and Carswell [47], drawing on the proximity compatibility principle, demonstrated that display formats consistent with users’ cognitive expectations yield superior monitoring performance. Therefore, the advantage of horizontal orientation in reducing reaction times can be attributed to its consistency with users’ natural left-to-right scanning patterns and its compatibility with feature-based attentional allocation. These factors jointly reduce search costs and promote more efficient perceptual processing, offering valuable guidance for the ergonomic design of dynamic visual displays in safety-critical environments.

### 5.3. Effects of Color-Coding Strategies Under Dynamic Multi-Level Alarm Conditions (RQ3)

We also analyzed the effects of cue number and cue type on ACC and RT under different experimental conditions. The results indicated that the number of color cues had a significant positive impact on both ACC and RT. As cue redundancy increased, participants were able to recognize alarm levels more quickly and accurately. This finding is consistent with the redundancy gain theory, which posits that multiple convergent signals enhance perceptual salience and reduce decision uncertainty [48]. One possible explanation is that redundant cues strengthen attentional guidance, making it easier to rapidly locate and differentiate alarm states. Prior studies in cognitive psychology have shown that convergent cues amplify perceptual contrast and facilitate visual search efficiency [49]. Another explanation is that increasing the number of cues helps reduce task complexity, especially in demanding recognition contexts. When alarm states are presented with multiple supportive cues, ambiguity decreases, thereby reducing cognitive load and accelerating decision-making. This interpretation aligns with Wickens [50], who demonstrated that providing multiple, consistent information channels reduces mental workload and improves performance in complex monitoring environments. Therefore, our findings indicate that increasing the number of color cues enhances alarm recognition by both reinforcing attentional guidance and alleviating the cognitive demands of decision-making, leading to higher accuracy and faster responses.

Interestingly, no interaction was observed between color cues and orientation, suggesting that these two factors exerted independent rather than synergistic effects: color cues improved accuracy and efficiency regardless of orientation, while horizontal orientation shortened reaction time regardless of cue presence. One possible explanation is that color cues and orientation may operate via distinct cognitive pathways. Color cues primarily leverage preattentive processing to direct attention toward semantically relevant information, whereas orientation effects are more closely linked to perceptual organization and habitual scanning strategies [51]. Because these mechanisms affect different stages of information processing, their influences appear largely additive rather than interactive. Similar independence between perceptual dimensions has been observed in prior visual search studies [43,44], which showed that separable features such as color and spatial arrangement often contribute individually to performance. Accordingly, the absence of an interaction effect in our study suggests that the reliability of color coding and the efficiency of horizontal orientation could be harnessed in parallel, with each factor maintaining its influence independently in dynamic alarm visualization tasks.

### 5.4. Theoretical and Practical Implications

Theoretically, the findings can be interpreted in relation to existing frameworks rather than as direct tests of those theories. The facilitation of foreground and label color cues aligns with attentional guidance theory, the performance benefits of cue redundancy are consistent with redundancy gain theory, and the orientation advantage reflects principles of spatial compatibility, as outlined in the proximity compatibility principle. The independence of effects between color cues and orientation also resonates with feature integration accounts, which posit that distinct perceptual channels exert additive rather than interactive influences. Together, these interpretations illustrate how established theories of attention and display design can converge in the context of dynamic alarm visualization.

Practically, our findings provide concrete guidance for designing DSBC-based alarm displays in safety-critical systems. For instance, in nuclear power plant interfaces, applying color cues to perceptually dominant elements such as the foreground segment representing core temperature can support faster identification of transitions into warning or critical states. Similarly, in chemical process control, color-coded labels on pressure or concentration thresholds can help operators verify alarm semantics without additional navigation. The advantage of horizontal orientation suggests that systems relying on left-to-right temporal interpretation (e.g., turbine output dashboards or load-following control panels) should prefer horizontal DSBCs to facilitate quicker semantic discrimination. In contrast, in domains like medical monitoring, where parameters are conventionally displayed vertically, vertical DSBCs may be used but should still incorporate prominent foreground or label cues to ensure clarity. Additionally, the observed redundancy gain implies that in high-workload settings like aviation, alarm recognition during time-critical phases (e.g., takeoff roll) can be improved by combining foreground with label cues. Together, these domain-specific strategies illustrate how hierarchical color cues and orientation choices can be operationalized to improve situational awareness and reduce cognitive load in real-world safety-critical environments.

### 5.5. Limitations and Future Research

This study has limitations that point to valuable directions for future research. First, the external validity of our findings may be bounded by the use of simplified DSBC stimuli and a student participant pool. Although these choices enabled controlled experimental isolation of key variables, future work should examine whether the identified perceptual advantages of specific color cues and horizontal orientation generalize to richer, multi-window interfaces and to domain experts in operational settings such as industrial control or aviation. Second, the scope of the examined cues presents another avenue for extension. While this study focused on color and orientation, other modalities (e.g., shape, auditory, or haptic signals) could interact with the observed mechanisms. Future studies investigating multimodal integration would provide a more comprehensive understanding of alarm recognition. Third, the complexity of the experimental design, involving the simultaneous, binary manipulation of multiple color attributes, presents inherent challenges for interpreting higher-order interactions. This interpretive challenge is compounded by the limited number of trials per specific condition. Although a power analysis (conducted using the “pwr” package in R [52,53]) confirmed that the overall design had adequate sensitivity to detect large effects (achieved power = 0.888 for *η*_p_^2^ = 0.14 at *α* = 0.05, exceeding the conventional threshold of 0.80 [32,54,55]), the few repetitions per condition per participant may limit the precision of individual-level estimates and the stability of complex interaction patterns. Therefore, future research could enhance robustness by employing simplified designs, increasing trial counts in focal conditions, or focusing on a targeted subset of theoretically critical factor combinations. Finally, the effectiveness of the tested color cues relies on conventional semantic associations (e.g., red-yellow-green). Consequently, cultural or industry-specific differences in color interpretation could moderate these effects. Future research should therefore explore alternative color conventions and culturally adapted palettes to determine the boundary conditions of the findings.

## 6. Conclusions

This study systematically examined the perceptual and cognitive effects of color cue application and orientation on users’ recognition of alarm semantic levels in dynamic stacked bar charts (DSBCs). The findings revealed three key conclusions. First, foreground and label color cues significantly enhanced recognition accuracy and efficiency, demonstrating that preattentive color signals effectively guide visual attention toward semantically relevant information. In contrast, background and scale line color cues contributed little to performance, underscoring the importance of selectively applying color to perceptually dominant chart elements. Second, horizontal orientation facilitated faster reaction times compared to vertical orientation without compromising accuracy, indicating that spatial alignment with natural left-to-right scanning patterns improves perceptual fluency and search efficiency. Third, increasing the number of color cues yielded cumulative benefits in both accuracy and speed, consistent with the redundancy gain principle, which posits that multiple convergent cues reduce uncertainty and cognitive load. However, excessive color application may risk perceptual overload, suggesting the need for careful cue optimization. No interaction was found between color cueing and orientation, indicating that they function through distinct cognitive mechanisms—color cues enhance attentional selection, while orientation influences spatial organization and perceptual sequencing. These results extend the applicability of attentional guidance, redundancy gain, and spatial compatibility theories to dynamic alarm visualization. Practically, the study provides empirical design guidance for optimizing DSBCs in safety-critical environments such as industrial control, aviation, and medical monitoring systems. Designers should prioritize color coding of foreground and label elements, adopt horizontal layouts in time-sensitive contexts, and apply redundant cues judiciously to balance visual salience and cognitive load. Furthermore, the proposed DSBC framework can be readily integrated into sensor-driven monitoring and decision-support systems, where real-time visualization of multidimensional sensor data is essential for early anomaly detection, situational awareness, and operational reliability.

## Figures and Tables

**Table 1:** Mean and standard deviation of accuracy (ACC) and reaction time (RT) (ms) for each level of the independent variables (N = 30).

Variables	Number of Trials	Accuracy (%)		Number of Trials	Reaction Time (ms)	
		Mean	SD		Mean	SD
Background Color Cue						
Presence	1920	0.929	0.257	1702	2487.635	989.355
Absence	1920	0.923	0.267	1707	2488.500	867.740
Foreground Color Cue						
Presence	1920	0.932	0.251	1698	2243.661	964.188
Absence	1920	0.920	0.272	1711	2730.618	827.110
Scale Line Color Cue						
Presence	1920	0.931	0.253	1721	2479.822	885.173
Absence	1920	0.921	0.270	1688	2496.475	974.372
Label Color Cue						
Presence	1920	0.932	0.251	1713	2468.841	1002.092
Absence	1920	0.920	0.272	1696	2507.488	851.550
Chart Orientation						
Horizontal	1920	0.926	0.263	1740	2327.388	818.859
Vertical	1920	0.927	0.261	1669	2655.584	1007.092

**Table 2:** Results of linear mixed effects regression analysis on reaction time.

Effects	ΔAIC	LLR χ^2^	*p* Value
Foreground Color Cue	−470	472.4467	<0.001
Background Color Cue	2	0.2137	0.644
Label Color Cue	−4	6.0828	<0.05
Scale Line Color Cue	0	1.3369	0.248

**Table 3:** Results of logistic mixed effects regression analysis on accuracy.

Effects	ΔAIC	LLR χ^2^	*p* Value
Foreground Color Cue	−3	5.0044	<0.05
Background Color Cue	0.74	1.2555	0.263
Label Color Cue	−3.05	5.0478	<0.005
Scale Line Color Cue	−1.53	3.5331	0.060

**Table 4:** Post hoc analyses of three-way interactions in RT based on nested linear mixed-effects models.

Effects	ΔAIC	LLR χ^2^	*p* Value
Foreground Color Cue × Background Color Cue × Scale Line Color Cue	−14	20.222	0.00015
Foreground Color Cue × Background Color Cue × Label Color Cue	3	0.085	0.9580
Foreground Color Cue × Label Color Cue × Scale Line Color Cue	−33	35.727	<0.001
Background Color Cue × Label Color Cue × Scale Line Color Cue	−3	11.274	0.0236

**Table 5:** Results of four orientation × color-cue (Foreground, Background, Label, Scale Line) two-way interactions on RT.

Effects	ΔAIC	LLR χ^2^	*p* Value
Orientation × Foreground Color Cue	−488	492.781	<0.001
Orientation × Background Color Cue	−3	6.595	0.037
Orientation × Label Color Cue	−3	7.239	0.027
Orientation × Scale Line Color Cue	2	1.710	0.425

**Table 6:** Results of four orientation × color-cue (Foreground, Background, Label, Scale Line) two-way interactions on accuracy.

Effects	ΔAIC	LLR χ^2^	*p* Value
Orientation × Foreground Color Cue	−1	5.065	0.097
Orientation × Background Color Cue	2.7	1.297	0.523
Orientation × Label Color Cue	−1.1	5.089	0.079
Orientation × Scale Line Color Cue	0.4	3.535	0.171

## Data Availability

The original contributions presented in this study are included in the article. Further inquiries can be directed to the corresponding author.
